# Distinct motivations to seek out information in healthy individuals and problem gamblers

**DOI:** 10.1038/s41398-021-01523-3

**Published:** 2021-07-26

**Authors:** Irene Cogliati Dezza, Xavier Noel, Axel Cleeremans, Angela J. Yu

**Affiliations:** 1 Centre for Research in Cognition and Neurosciences, ULB Neuroscience Institute, Université Libre de Bruxelles, Bruxelles, Belgium; 2 Department of Experimental Psychology, Faculty of Brain Sciences, University College London, London, UK; 3 The Max Planck UCL Centre for Computational Psychiatry and Ageing Research, University College London, London, UK; 4 Department of Experimental Psychology, Ghent University, Ghent, Belgium; 5 Faculty of Medicine, Université Libre de Bruxelles, Bruxelles, Belgium; 6 Department of Cognitive Science, University of California San Diego, San Diego, USA

**Keywords:** Addiction, Human behaviour

## Abstract

As massive amounts of information are becoming available to people, understanding the mechanisms underlying information-seeking is more pertinent today than ever. In this study, we investigate the underlying motivations to seek out information in healthy and addicted individuals. We developed a novel decision-making task and a novel computational model which allows dissociating the relative contribution of two motivating factors to seek out information: a desire for novelty and a general desire for knowledge. To investigate whether/how the motivations to seek out information vary between healthy and addicted individuals, in addition to healthy controls we included a sample of individuals with gambling disorder—a form of addiction without the confound of substance consumption and characterized by compulsive gambling. Our results indicate that healthy subjects and problem gamblers adopt distinct information-seeking “modes”. Healthy information-seeking behavior was mostly motivated by a desire for novelty. Problem gamblers, on the contrary, displayed reduced novelty-seeking and an increased desire for accumulating knowledge compared to healthy controls. Our findings not only shed new light on the motivations driving healthy and addicted individuals to seek out information, but they also have important implications for the treatment and diagnosis of behavioral addiction.

## Introduction

Recent advancements in neuroscience have shown information-seeking to be an essential aspect of human cognition that supports healthy decision-making and goal-directed processing [1–8]. Information-seeking is often contraposed to the human tendency of maximizing immediate benefits (i.e., reward-seeking). A decision-maker who is trying to find the best restaurant in town may try out all the different available options in order to obtain information on the potential benefits of each restaurant, but this information search may be costly or result in unpleasant experiences.

Yet, healthy humans finely balance the urge for immediate reward vs. longer-term information gain during repeated choice behavior, thus negotiating an exploration–exploitation trade-off [4, 6, 9]. On the contrary, in certain psychopathologies such as behavioral addiction [10] resolving this tension is highly compromised resulting in reduced information-seeking [11]. Previous studies have suggested that a desire for *novelty* [1, 12–16] and a general drive to seek out knowledge (*general information* [17, 18]), may both drive human information-seeking behavior. However, there has been no study that systematically analyzes the relative importance of these two factors in healthy humans, nor how these information-seeking systems might be altered in behavioral addiction.

While novelty is only associated with a completely novel item, a general desire for knowledge can promote the exploration of an option beyond the first encounter. These two motivational factors are however highly related, since the potential for general knowledge gain and a novelty bonus can be easily mistaken for one another as statistically significant explanatory factors. However, these two motivational factors seem to rely on different neural regions in the brain, with novelty-seeking expressed in midbrain dopaminergic regions [12, 13, 19, 20] and general information seeking in prefrontal regions [21–23]. Here, we state that the distinction between novelty-seeking and general information-seeking is essential to understand the underlying motivations to seek out information in healthy and addicted individuals.

For example, evidence for general information-seeking has come from variants of sequential learning and decision-making tasks (e.g., the bandit tasks [4, 6, 8, 24]). This may leave the possibility that general information-seeking is more important for scenarios in which repeated choices are necessary such as during learning or planning, while novelty-seeking might be more relevant for single-stage decisions or early stages of learning [13, 15]. In addition, impaired information-seeking in addictive disorders [11, 25] has been explained as a general reduction in the desire to reduce uncertainty about the environment. However, these impairments might be equally explained as a reduced desire for exploring novel opportunities or engaging in novel behavioral patterns. This distinction is crucial for behavioral addiction—of which gambling disorder is a prototype [10]. If reduced information-seeking is caused by a reduced motivation specifically for exploring novel options, this could explain why pathological gamblers exhibit perseverance in behavioral routines despite the negative consequences associated with them (e.g., financial loss [10]), but at the same time, they still prefer choices associated with high uncertainty about reward outcomes (e.g., gambling games such as gaming machines or blackjack [26, 27]). Insight into the distinction between novelty-seeking and general information-seeking is therefore particularly relevant for understanding addictive behaviors, as well as potentially developing better diagnostic tools or clinical treatments.

Here, we explicitly compare novelty-seeking and general information-seeking in a modified version of the bandit task, which makes it possible to dissociate the relative contribution of expected reward, novelty, and general information as motivating factors in choice behavior. In particular, choices in our task are reward-driven if participants chose options associated with the highest experienced rewards, novelty-driven if participants chose options that had never been experienced in the previous trial history, and general information-driven if participants chose options that had been previously encountered but imperfectly explored (“Method and material”). We also implement a reinforcement-learning type model to quantitatively separate out the importance of these three factors in driving human choice behavior. In addition to healthy controls (HCs), we include a sample of individuals with gambling disorders (PGs). This allows us to investigate the relative contribution of general information-seeking and novelty-seeking in behavioral addiction.

## Methods and material

### Participants

Forty (40) unmedicated PGs (mean age = 30.1, 4 females) and twenty-two (22) HCs (mean age = 29.0, 4 females) were recruited from the local communities (Table 1 and Supplementary results). The sample size of both groups was based on previous studies [6, 28]. Gamblers were selected among those who were gambling at least once per week, while HCs were those without gambling experience in the year preceding experimental participation (Table 1 and Supplementary results). Subjects were compensated for the time spent in the study.

**Table 1:** Demographic information.

	PGs *n* = 40	HCs *n* *=* 22	Test statistic
Gender (M/F)	36 | 4	18 | 4	*p* = 0.601
Age	30.1(9.3)	29.0(6.6)	*p* = 0.982
Years of education	14.7(2)	16.2(2.2)	*p* = 0.037*
IQ (WAIS block)	8.4(2.6)	9.3(1.9)	*p* = 0.131
Gambling severity (CPGI)	8.8(6.1)	0	*p* < 10^−10^*
Alcohol use (AUDIT)	4.6(3.9)	5.3(3.1)	*p* = 0.48
Drug use (DAST)	0.225(0.423)	0.227(0.429)	*p* = 0.992
Smoking dependence (FTND)	*n* = 4	*n* = 1	NA
Memory capacity (WAIS)	10.3(3.5)	9.7(4.1)	*p* = 0.483
Attentional control (ACS)	35.4(9)	37.5(7)	*p* = 0.312
Depression (BDI)	5.6(4.9)	4.2(4.8)	*p* = 0.137
Anxiety (STAI-S)	35.1(10.9)	37.9(9.5)	*p* = 0.173
Anxiety (STAI-T)	39.6(12.4)	43.1(11)	*p* = 0.2
Positive mood (PANAS)	35.4(6.3)	36.3(5.3)	*p* = 0.701
Negative mood (PANAS)	21.1(7.9)	19.8(4.8)	*p* = 0.808

Mean and standard deviations are shown for each measure. For each comparison, we ran a two-sampled *t* test, except for gender comparison where the chi-squared test was used. The two groups differ only in terms of gambling severity (with no gambling problems reported in the control group) and years of education, as often reported in the literature [28] (years of education did not correlate with any of the behavioral measures considered in this study, and removing PGs with fewer years of education did not change the main results reported in the text). WAIS IV-Wechsler Adult Intelligence Scale (the block-design component of the WAIS is the subtest that best predicts performance IQ [58]).

CPGI Canadian problem gambling index, AUDIT alcohol use disorders identification test, DAST drug abuse screening test, FTND Fagerström test for nicotine dependence, ACS attentional control scale, BDI beck depression inventory, STAI-S state version of the state-trait anxiety inventory, STAI-T trait version of the state-trait.

### Behavioral task

**Fig. 1:**
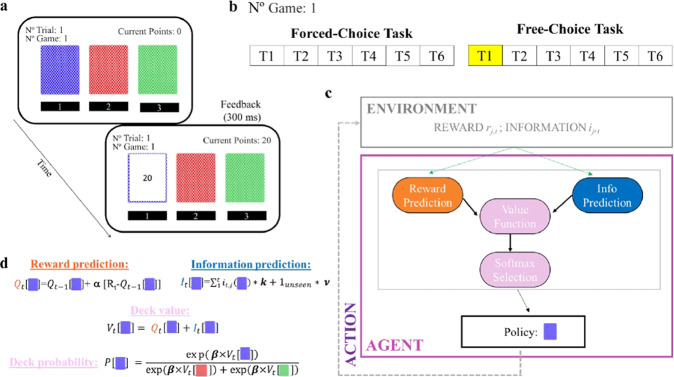
Behavioral task and RL model. **a** On each trial, participants made choices among three decks of cards. After selecting a deck, the card flipped and revealed the points earned, between 1 and 100 points. Participants were instructed to attempt to maximize the total points earned by the end of the experiment. **b** On each game, participants played a forced-choice task (six consecutive trials) followed by a free-choice task (variable between 1 and 6 trials) on the same three decks. Subjects earned points only in the free-choice task. **c** On each trial, the novelty-knowledge RL (nkRL) model computes an option value function according to both experienced reward and information associated with each option, then the model generates a choice by passing the option values through a softmax function. **d** For each chosen option, nkRL uses a delta rule to update the reward prediction (*α* parameterizes the learning rate) and updates information prediction as to the sum of general information (total number of times an option has been chosen) and a novelty term. The general information term describes the level of general information participants have about the selected option, while the novelty bonus is assigned to options the outcome of which has never been experienced in previous trials. Reward and information predictions are then combined into an overall action value, which is combined across options through the softmax function (whose randomness is parameterized by the inverse-temperature parameter *β*). Model parameters are shown in bold.

Participants performed 162 games of a decision-making task [6] that makes it possible to dissociate the influence of reward and information on sequential choices [4] (Fig. 1a and Supplementary methods). On each trial, choice options were displayed on the screen as 3 decks of cards. Selecting a deck revealed a card associated with a certain number of points. Each game consists of two phases (or tasks): participants were initially instructed about which option to choose from on each trial (forced-choice task; Fig. 1b) for six consecutive trials, after which they were free to choose from any of the options (free-choice task; Fig. 1c) so as to maximize their total gain. In the forced-choice task, participants needed to choose a preselected deck that was highlighted in blue. In the free-choice task, on the contrary, they were free to select the deck of their choice. The number of free-choice trials varied from 1–6 trials and was inverse-exponentially distributed, such that subjects were most frequently allowed to make 6 free choices. Participants played the task for about 1 h.

When selected, each deck provided a reward (from 1 to 100 points) generated from a truncated Gaussian distribution with a fixed standard deviation of 8 points, and then rounded to the nearest integer. Participants were instructed that the total gain (i.e., the total points accumulated across trials) was converted to a monetary payoff at the end of the experiment where every 60 accumulated points correspond to 0.01 euros. The generative mean for each deck was set to a base value of either 30 or 50 points and adjusted independently by ±0, 4, 12, or 20 points (i.e., the generative means ranged from 10 to 70 points) with equal probability, to avoid the possibility that participants might be able to discern the generative mean for a deck after a single observation. The generative mean for each option was stable within a game but varied across games. The generative mean of the three decks had the same value in 50% of the games (*Equal Reward)* and different values (*Unequal Reward*) in the other 50% of the games. In the Unequal Reward condition, the generative means differed so that two options had the same *higher* reward values compared to the third one in 25% of the games (*High Reward*), and in 75% of the games, two options had the same *lower* reward values compared to the third one (*Low Reward*). The appearance of the reward conditions was randomized, as were the assignments of which two options had the same generative mean within each game (in the Unequal Reward games).

In the free-choice task, participants could either select the options from which they saw the highest number of points drawn from or they could instead explore the other two alternatives. On trials when participants explore additional alternatives, they can either choose at random (undirected or random exploration) [4], direct their exploration toward a novel option (novelty-seeking), or distribute their exploration among alternatives inversely proportional to how frequently they have been seen in the past (general information-seeking). In order to dissociate among these factors, we implemented two conditions in the forced-choice task [4]. Participants were either forced to choose each of the three decks 2 times (*Equal Information*), or to choose one deck four times, a second deck 2 times and the third 0 times (*Unequal Information*). In the latter condition, the “0 time” deck is perceptually familiar to participants (the stimulus is presented at the beginning of the game) but its reward distribution is novel to participants. While only the “0 time” deck is completely novel, the “2 times” deck should be relatively more information-rich than the “4 times” to the participants. As generative means were equal across options in 50% of the total games, the generative means of the decks that had been sampled 2 times were equal to those of the decks that had been sampled either 4 times or 0 times decks in 50% of Unequal information trials, while it had lower or higher values in the rest of the trials. This assures that the reward associated with this option was balanced across trials. 50% of the games were assigned to the Unequal information condition. The order of card selection was randomized in both information conditions, as was the occurrence of the equal and unequal information conditions.

Considering only the first free-choice trial (the trial where reward and information are least correlated [4]; Supplementary methods), we then define three types of behaviors, corresponding to three distinct motivational factors: (1) *Novelty-seeking* refers to choosing the novel, never-seen option in the Unequal Information condition; (2) *General information-seeking* refers to choosing partially informative options sampled twice in the Unequal Information condition—these options are still informative when explored but not completely novel; (3) *Reward-seeking* refers to choosing options associated with the highest gain. In addition, we define a fourth behavior—*undirected exploration*—which refers to choosing options associated with the lowest gain in the Equal Information condition, as this type of choice is neither driven by reward nor by information.

### Computational model

We assume that humans behave according to both reward- and information-related internal beliefs/motivation when performing the above decision-making task [6]. We formalize this using a reinforcement-learning (RL) type computational model (Fig. 1c). In order to investigate the nature of information valuation in HCs and PGs, we implement a novel computational model that we term the “novelty-knowledge RL” (nkRL) model. As in a previously proposed variant, nkRL learns reward expectations $Q_{t + 1,j}\left( c \right)$ using a delta learning rule [29] (Eq. (S1) and Fig. 1d) where $Q_{t + 1,\,j}\left( c \right)$ is updated each time a new reward is experienced from option c. Next, the value of an option $V_{t,\,j}\left( c \right)$ is determined by combining reward expectations and information evaluations (Eq. (S3) [6] and Fig. 1c, d). Contrary to previous variants, nkRL specifically dissociates information evaluation into two terms: novelty and general information-seeking. The resulting choice value is:

1 $$V_{t,\,j}\left( c \right) = Q_{t + 1,\,j}\left( c \right) + \mathop {\sum}\limits_1^t {i_{t,\,j}\left( c \right) \ast k + 1_{{{{\mathrm{novel}}}}} \ast \nu }$$

where $Q_{t + 1,j}\left( c \right)$ is the expected reward value on trial *t* in-game *j* for choice *c* (computed using Eq. (S1)) and the last two terms represent general information and novelty, respectively. In particular, $\mathop {\sum}\nolimits_1^t {i_{t,j}\left( c \right)}$ is the cumulative information about option *c* acquired through trial t ($i_{t,j}$ is 1 if selected on trial *t*, or 0 otherwise). *k* is the *knowledge* parameter that defines the weight toward previously acquired information. We acknowledge that this parameter does not distinguish between attraction/repulsion of cumulative knowledge and repulsion/attraction of incremental knowledge gain. $1_{{{{\mathrm{novel}}}}} \ast \nu$ captures the value associated with *novelty*, where 1_novel_ is a Kronecker delta function that evaluates to 1 when *c* has never been seen in the current game and 0 otherwise, and the parameter *ν* quantifies the value associated with novelty. As in previous algorithms in artificial intelligence, the novelty bonus is incorporated as optimistic initialization to the starting value of novel options [30]. Finally, we assume choices are made via a softmax function of ${{{\mathrm{V}}}}_{{{{\mathrm{t}}}},{{{\mathrm{j}}}}}\left( {{{\mathrm{c}}}} \right)$ [31] (Eq. (S2)), where options with a higher choice-value would result in a higher probability to determine the choice on that trial. As we assume that participants’ choices are not deterministic, decision noise is entered into the softmax function by adding the inverse temperature parameter *β* (Fig. 1d). NkRL can shed light on the processes that underpin information valuation in both HCs and PGs by distinguishing the effects of reward-seeking and information-seeking on choices (*β* vs. *k*, *ν*), and of novelty and knowledge on information-seeking (*ν* vs. *k*). The model’s parameters are estimated by fitting nkRL to trial-by-trial participants’ free choices (Supplementary methods). NkRL model will be compared to additional models to determine which model best-described participants’ choices in our task. Model comparison was carried out by computing the Bayesian Information Criterion (BIC; Supplementary methods), for which the lower the value, the better the model is in explaining the data.

## Results

### Model-free results

#### Novelty-seeking in HCs and novelty-failure in PGs

**Fig. 2:**
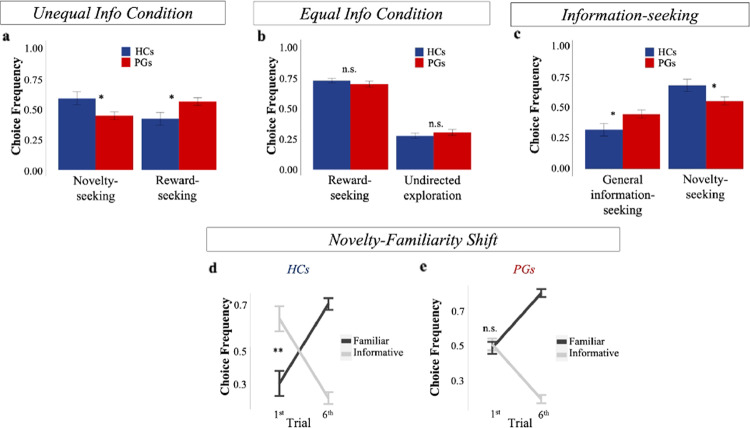
Model-Free analysis. **a** Frequency of making *novelty-seeking* and *reward-seeking* choices over the total number of novelty-seeking and reward-seeking trials in the first free-choice trial of the Unequal Information condition (i.e., when options are sampled unequally during the forced-choice task; Unequal Info Condition in the figure). Novelty-seeking choices decreased and reward-seeking choices increased in PGs compared to HCs. **b** Frequency of engaging in *reward-seeking* and *undirected exploration* in the first free-choice trial of the Equal Information condition (i.e., when options are sampled equally during the forced-choice task; Equal Info Condition in the figure). No difference was observed between the two groups. **c** Frequency of engaging in *novelty-seeking* and *general information-seeking* over the total number of information-seeking trials in the first free-choice trial of the Unequal Information condition: PGs have reduced information-seeking toward novel options (*novelty-seeking*) relative to information-seeking toward options selected twice in the forced-choice task (*general information-seeking*). **d** HCs showed a novelty-familiarity shift: increased preference toward informative options in the first free-choice trial, and increased preference for familiar alternatives in the last free-choice trial. **e** PGs showed no preference between informative and familiar options in the first free-choice trial, but a significant preference toward familiar options on the last free-choice. In all the figures, error bars represent the standard error of the mean (sem).

We first examined how HCs and PGs compare the influence of reward and information on choice behavior. We focus on the Unequal Information condition (equal information games have no informative options) and the first free-choice trial, the one trial where we can be sure that information and experienced reward are uncorrelated (Supplementary methods [4]). We consider a trial to be *novelty-seeking* if the participant selects the novel option, and *reward-seeking* if the participant selects a previously experienced option with the higher empirical mean (regardless of whether it was seen twice or four times). For each subject, we computed the relative frequency of novelty-seeking trials and of reward-seeking trials over the total number of novelty-seeking and reward-seeking trials. We then entered these values into a mixed-effects logistic regression model predicting the choice type (novelty-seeking, reward-seeking), with group (PGs, HCs), reward condition (low reward, high reward), and their interaction as fixed effects, and subject as random intercepts (1|Subject). This standard random intercept model had lower BIC (6076.5) compared to a full random coefficient model (with random intercepts and slopes: BIC = 6110.2). First, consistent with previous studies using the same experimental design on healthy subjects [6, 9], we found a main effect of reward (beta coefficient = −0.824 ± 0.104 (SE), *z* = −7.90, *p* < 10^−3^), with novelty-seeking generally more common in the Low Reward condition. More interestingly, we found a significant fixed effect of group (beta coefficient = 0.643 ± 0.268 (SE), *z* = 2.4, *p* = 0.016), with PGs engaging in less novelty-seeking and more in reward-seeking behavior (Fig. 2a). The interaction between group and reward condition was not significant (beta coefficient = −0.144 ± 0.132 (SE), *z* = −1.093, *p* = 0.274), suggesting that the two groups did not differ in the way the reward conditions affected choice behavior.

Interestingly, PGs and HCs show comparable choice behavior when choices were equally informative (Equal Information condition, Supplementary results and Fig. 2b). This suggests that differences between the two groups were only present when choices were associated with different levels of information. In addition, the shift in preference from more informative options (when subjects chose the option sampled the least number of times) early on in the free-choice task to more familiar options (when they chose the option sampled the most number of times) later on was smaller in PGs than HCs (Supplementary results). Lastly, a “novelty-familiarity” shift was apparent in HCs (they preferred novel options in the first free choice trial) but absent in PGs who preferred novel options and familiar options equally on trial 1 (Fig. 2d, e).

#### PGs have reduced preference for novelty but not for general information

The above analyses yielded hints that PGs have reduced preference specifically for novelty. To test this suggestion, we calculated the number of trials in which participants engaged in novelty-seeking and in general information-seeking (partially informative options sampled twice during the forced-choice task) and divided them by the total number of novel and general information trials to obtain their relative frequencies (i.e., we excluded trials in which the subject chose the option that was selected 4 times during the forced-choice task). If alterations in PGs’ behavior are not specific to novelty, we should also expect to find a lower selection of options experienced twice during the forced-choice task. Results showed that while PGs chose the novel option less often than HCs (*p* = 0.015; Fig. 2c) on the first free-choice trial in the Unequal Information condition, PGs chose the partially informative option (seen twice) *more often* (*M* = 0.446, SD = 0.21) compared to HCs (M = 0.32, SD = 0.239; Wilcoxon Signed Rank test, *p* = 0.015; Fig. 2c), suggesting that PGs specifically shy away from novelty-seeking. This was also the case when restricting the analysis to trials in which the 3 decks had the same generative means (for partially informative options—*M*_PGs_ = 0.46; *M*_HCs_ = 0.319; Wilcoxon Signed Rank test, *p* = 0.015). As an additional check, we constructed a mixed logistic regression model to predict choice type (partially informative option, familiar option, i.e., excluding novel option trials) with group (PGs, HCs) as fixed effect and subject as random intercept term (1|Subject; this model had lowest BIC compared to a model with random intercepts and slopes), and found no effect of group (beta coefficient = 0.011 ± 0.088 (SE), *z* = 0.12, *p* = 0.905), additionally suggesting no decrease in general information-seeking in PGs compared to HCs. We further examine this point in the next section.

### Model-based results

#### HCs have increased novelty bonuses, while PGs have increased knowledge parameter

**Fig. 3:**
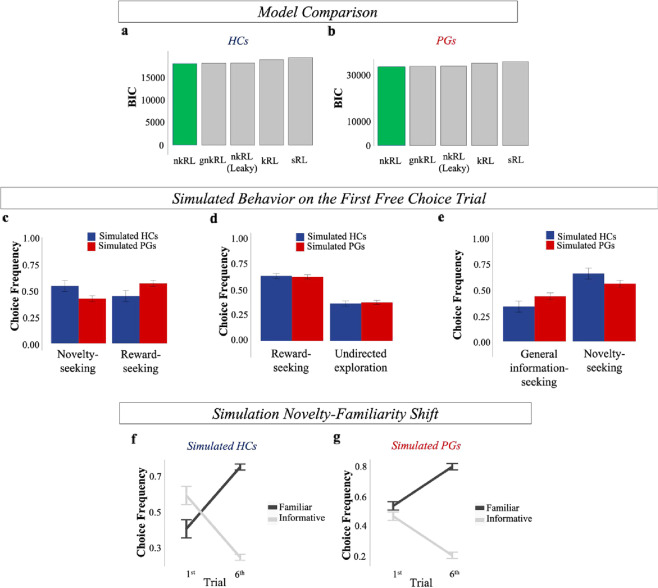
Model Comparison and nkRL simulations. BIC comparison of the 5 RL models in HCs (**a**) and PGs (**b**). The comparative fit is based on the sum of individual BIC computed by fitting each model to participants’ free choices. In both groups, the novelty-knowledge RL model (nkRL, in green) better explains participants’ behavior compared to a leaky novelty-knowledge RL model (leaky nkRL), a knowledge RL model (kRL), a standard RL model (sRL), and a gamma novelty-knowledge RL model (gnkRL). By using the estimated individual parameters, simulations of nkRL in the first free choice trial reproduced the empirically observed decrease in novelty-seeking in PGs (Unequal Information condition, (**c**)), comparable choice behavior when choices are equally informative (Equal Information condition, (**d**)), an increase of preference for partially informative options (general information-seeking, (**e**)). **f** nkRL correctly predicts the novelty-familiarity shift in the healthy sample, (**g**) and its absence in the PG group. Error bars: sem (color figure online).

In order to further elucidate the mechanisms underlying information-seeking in HCs and PGs, we turn to model-based analyses. Here, we propose a novel reinforcement learning-type model that we call “novelty-knowledge RL” (nkRL, “Methods and material”). We first ran a model comparison analysis (Supplementary methods) and observed that nkRL was better able to explain participants’ behavior compared to the following models: a standard RL (sRL) model [29], where only reward predictions influence choices; a knowledge RL (kRL) model [6], which linearly combines reward and information associated with options without explicitly decomposing information into novelty and general information; leaky nkRL, where information accumulation across trials proceeds in a leaky fashion; gamma nkRL (gnkRL), where information is measured sub- or super-linearly in the number of observations (Fig. 3a, b; Supplementary results).

We then utilized nkRL to better investigate the process underlying the differences in information-seeking between PGs and HCs. We first simulated nkRL, using the individually fit parameters, to verify that the model was able to replicate key behavioral patterns observed in the data. As shown in Fig. 3, nkRL is able to qualitatively reproduce key behavioral patterns observed in both groups, including reduced novelty-seeking in PGs compared to HCs (Fig. 3c), comparable choice behavior when choices are equally informative (Fig. 3d), an increase of preference for partially informative options (general information-seeking, Fig. 3e), and the absence of a novelty-familiarity shift in PGs (Fig. 3g).

**Fig. 4:**
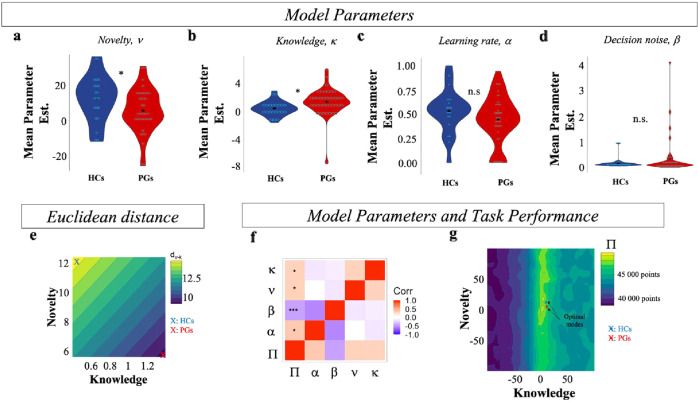
*nkRL* parameters and information-seeking modes. Model fit on all free-choice trials revealed a decrease in the novelty parameter *ν* in PGs compared to HCs (**a**), while the knowledge parameter *κ* was higher in PGs compared to HCs (**b**). Learning rate *α* (**c**) and decision noise *β* (**d**) did not differ between the two groups. **e** The Euclidean distance *d*_*v-k*_ between κ and ν was larger in HCs (in blue) than in PGs (in red). **f** Correlation matrix between nkRL model parameters and task performance *Π*. *P*-values are corrected for multiple comparisons (FDR). Both *ν* and *κ* positively correlated with Π. Only correlations between *Π* and model parameters are reported. **g** Performance *Π* across *ν* and *κ* parameter space. The averaged values of ν and κ for HCs are shown in blue, while in red for PGs. The two averaged values are expressed closer to the two optimal modes (in yellow) (color figure online).

Next, we performed parameter comparison analyses to examine which component of the decision-making process may be responsible for the behavioral pattern observed in PGs. We first performed a parameter recovery analysis to estimate the degree of accuracy of the fitting procedure (Supplementary results; Fig. S1). We were able to recover all the parameters with high accuracy (all *r* > 0.8). We then compared the parameter estimates between the two groups. A Wilcoxon Signed Rank Test showed smaller novelty parameter *ν* in PGs (*M* = 5.58, SD = 12.11) compared to HCs (*M* = 12.43, SD = 12.91, *p* = 0.0416; Fig. 4a), while the knowledge parameter *k* was higher in PGs (*M* = 1.38, SD = 2.01) compared to HCs (*M* = 0.43, SD = 1.04, *p* = 0.0017; Fig. 4b). In line with our model-free results, these results suggest that PGs have reduced information-seeking for novelty, but not for knowledge accumulation. We further explored this result by entering parameter (*ν*, *k*) and group (HCs, PGs) in a two-way repeated measure ANOVA in a non-parametric setting using aligned rank transformation (e.g., ARTool package in R, http://depts.washington. edu/madlab/proj/art/; [32]). This revealed an effect of group (F(1,58) = 10.06, *p* *=* 0.002), an effect of parameter (F(1,58) = 40.19, p < 10^−3^) and an interaction between group and parameter (F(1,58) = 18.13, *p* < 10^−3^). These results seem to confirm that the decrease in information-seeking in PGs is due to a failure in either computing or utilizing a novelty bonus and to increased preference for previously encountered alternatives. Interestingly, the two parameters interacted in a way such that their relative difference was higher in HCs compared to PGs. To further investigate this, we computed the Euclidean distance between *ν* and *k* (d_v-k_) in the parameter space. Results showed that *d*_*v-k*_ was larger in HCs (*M* = 14.9, SD = 9.3) than in PGs (*M* = 9.2, SD = 7.5) *p* = 0.034 (Fig. 4e). By simulating nkRL with low novelty parameter (i.e., small *d*_v-k_) and high novelty parameters (i.e., large *d*_v-k_), the model was able to predict the behavioral pattern observed in PGs and HCs, respectively (Supplementary results; Fig. S2).

Lastly, PGs and HCs did not differ in either learning rate α or softmax parameter *β* (*p* < 0.2; Fig. 4c, d) suggesting that the behavioral patterns observed in PGs are not related to learning alterations, or due to an increase/decrease of random stochasticity in choice distribution. This latter result additionally confirms that exploratory impairments in PGs are specifically driven by novelty-related information valuation without affecting other undirected or unexplained exploratory components (e.g., softmax parameter). Overall, the model-based analyses suggest that HCs are specifically driven by novelty during exploratory behavior (*d*_*v-k*_ is larger and in the direction of high novelty bonus; Fig. 4e), while in gamblers the importance of novelty is reduced and the importance of knowledge accumulation is enhanced, resulting in a smaller distance between the two parameters.

#### Predicting pathological gambling from model parameters

In the previous section, we showed that PGs give higher weights to the knowledge parameter *k* and lower weights to the novelty parameter *ν* compared to HCs. Here, we check whether the values assigned to these two parameters can predict whether a participant is assigned to the PG group or HC group. To do so, we enter the values of both *k* and *ν* parameters in a logistic regression model predicting group (HCs = 0; PGs = 1). Results show that both *k* (beta coefficient = 0.569 ± 0.219 (SE), z = 2.61, *p* = 0.009) and *ν* (beta coefficient = −0.082 ± 0.031 (SE), *z* = −2.63, *p* < 10^−3^) predict group, with higher *k* parameter and lower *ν* parameter predicting PG group. We also run the same analysis but predict the gambling severity scale (CPGI—collapsing PGs’ and HCs’ scores in one variable). Results show that both *k* (beta coefficient = 1.025 ± 0.475 (SE), *z* = 2.16, *p* = 0.035) and *ν* (beta coefficient = −0.172 ± 0.066 (SE), *z* = −2.59, *p* = 0.0121) predict gambling severity, with higher knowledge parameter and lower novelty parameter predicting higher gambling severity.

#### HCs and PGs adopt distinct information-seeking modes

Previous analyses show that altered information-seeking in PGs compared to HCs is due to a decreased difference between *ν* and *k*, with PGs giving higher weight to knowledge accumulation and reduced weight to novelty, and this altered pattern predicts PG group as well as gambling severity. Here, we analyze how this particular pattern might affect PGs’ reward accumulation performance in the task. We define task performance as the sum of points earned on free-choice trials, summed across games. Our results show no difference in task performance (*Π*) between PGs and HCs throughout the task (all *p* > 0.05). We then correlated participants’ *Π* with the estimated model parameters for each subject in both groups. We entered *Π* and model parameters into a correlation matrix where *p*-values were corrected for multiple comparisons using false discovery rate correction (FDR [33]). Results show that both having increased novelty parameter and increased knowledge parameter relate to higher performance in the task (points earned; *p* < 0.05; Fig. 4f). This seems to suggest that high novelty and high knowledge parameters are equally helpful for yielding high performance in our task. In addition, no significant correlation was found between *Π* and the distance *d*_*v-k*_ (*p* = 0.096).

The above results seem to suggest that having a large (high novelty and low knowledge) or small (increased knowledge and decreased novelty) distance *d*_*v-k*_ yields good performance in the task. We further simulated the nkRL model with different settings of knowledge and novelty parameters, while keeping constant both alpha and beta parameters, to see whether there are indeed two different modes that yield good performance in the task. We computed Π for each simulation and we plotted it in the parameter space. Results show that two modes give high performance (Fig. 4g): one mode with high novelty and low knowledge parameters (*ν* = 19.02; *κ* = 5.37, *Π* = 48,835 points) and a second mode with similar values for knowledge and novelty parameters (*ν* = 2.55; *κ* = 2.97, *Π* = 49,251 points). Interestingly, average estimated values of ν and κ for the two groups are close to the two locally optimal modes. These results not only suggest that the differences between HCs and PGs’ information-seeking behavior correspond to adopting two alternative modes of adaptive behavior for the task, but that reward feedback from the task would not be effective for shifting either group’s behavior to the alternative local optimum.

## Discussion

In this study, we adopted behavioral, self-reported, and computational measures to investigate the underlying motivations to seek information in healthy and addicted individuals. We focus on gambling disorder, a form of addiction without the confound of substance consumption [34] and characterized by compulsive gambling [10]. We found that HCs and PGs adopt distinct information-seeking modes, closely related to the two locally optimal modes that yield good task performance. HCs’ information-seeking behavior appears mostly driven by novelty-seeking (choosing options which reward distribution was novel to participants) with little effect of knowledge accumulation (choosing known but imperfectly explored options). To the contrary, PGs exhibit enhanced general knowledge accumulation and reduced novelty-seeking compared to HCs. This pattern was also reflected in the model parameters and was predictive of gambling severity and membership in the PG group. Our findings not only shed new light on the motivations driving healthy and addicted individuals to seek out information, but also have important implications for the treatment and diagnosis of behavioral addiction.

One possible interpretation of our results in regard to the difference between PGs and HCs is that novelty-seeking may be particularly important for human wellbeing and mental health, and the relative reduction of novelty-seeking may underlie the pattern of maladaptive behavior in problem gamblers compared to HCs. The link between novelty-seeking and wellbeing has been already suggested in previous research [35], where novelty-seeking relates to positive affect in a bidirectional manner [36]. More research is however needed to better understand the specific role novelty-seeking plays in human wellbeing and mental health. Nevertheless, novelty-seeking seems to have an adaptive role as it is expressed both in animal models and motivates exploration in artificial agents. For example, animals can learn to press a key only for the sake of poking the head into a new compartment [37] or to guarantee the delivery of novel visual stimuli [38]. To encourage exploration in artificial agents a fictive reward bonus is given to novel options [30, 39], and similar heuristics seem to be adopted by the human brain [13]. Novelty biases therefore might be crucial for quickly understanding changing environments, as when novel options are available for selection. The increased weight PGs give to previously chosen options is consistent with a reduced novelty bonus (both reflecting unfamiliarity aversion), and might be a compensatory mechanism that arises in addictive behaviors. It is worth noting that while both modes of behavior make it possible to achieve good performance in our experimental task, in real life one may prove more maladaptive than the other in many situations. In addition, the benefit of novelty-seeking behaviors may also depend on the type of environment people are invited to explore. For example, novelty-seeking behaviors in dangerous environments might prove maladaptive and may lead to addictive behaviors in certain cases [40]. Future work is needed to investigate these issues further.

Another possible interpretation of our finding is that there is a single underlying pattern of alteration in the brain structure of PGs that affect both novelty-seeking and general information-seeking. Information-seeking behaviors are controlled by an interconnected cortico-basal ganglia network [41]. Previous studies [12, 13, 19–23] seem to support the dissociation of these two motivational factors within this network. Further work however is needed to individuate how the neural markers for novelty and general information interact within the information-seeking network and can produce the altered behavioral pattern observed in PGs.

An interesting implication of our findings is that regardless of the provenance of the alternative pattern of information-seeking in PGs compared to HCs, this altered behavior may be useful for developing novel diagnostic tools and even novel treatments for this pathology. First of all, our findings potentially suggest a novel method for identifying individuals with behavioral addiction, that is, reduced novelty drive and increased general information accumulation. However, further work is needed to demonstrate the validity of this behavioral marker and compare its role relative to other biomarker candidates [42]. Additionally, despite this behavioral pattern seeming to predict membership in the PG group as well as gambling severity, it is unclear whether these results can be generalized to different task settings (e.g., with larger rewards) or different samples of people. Second of all, novel theories on the pathophysiology of this disorder suggest that the resolution of reward uncertainty present in gambling games creates the capacity for addiction [26, 27, 43]. Our findings can help clarify why addictive behaviors are characterized by reduced information-seeking [11], and yet the source of addiction involves resolving uncertainty. Interestingly, reward uncertainty in addictive behaviors hijacks the dopaminergic system [27, 43, 44], as do drugs in substance addiction [45]. Given that novelty-seeking relies on the functioning of the midbrain dopaminergic system [13, 46, 47], this behavior may compete with responses towards reward uncertainty. In other words, reduced novelty-seeking might be a signature of this hijacking process.

Our study however does not rule out the possibility that neurophysiological alterations in the brain could pre-date or even induce problem gambling. In particular, it might be possible that individuals who exhibit the “reduced novelty-seeking and increased knowledge accumulation” mode may be more predisposed for developing (behavioral) addiction. When addictive behaviors arise, the reduced ability to adopt novel behavioral patterns may freeze their decision processes and trap them into the same behavioral routines. Reduced novelty-seeking might therefore explain why addicted individuals are trapped in the same behavioral routines despite the negative consequences associated with them (e.g., financial loss [10]).

On an additional note, novelty seems to compete with conditioned drug rewards [48]. This suggests that boosting novelty-seeking behavior may compete with the addictive stimuli and reduce the impact of addiction. Therefore, novelty-seeking might be introduced or induced in current treatments for addictive behaviors [49, 50]. Additionally, research has failed to identify the main mediators involved in cognitive and behavioral training aimed at reducing the attractiveness of addiction-related cues [51]. These types of interventions may directly or indirectly promote novelty-seeking behavior. Further work however is needed to test whether our findings can be extended to effective clinical interventions: in particular, whether the above behavioral procedure modifies the relationship between novelty-seeking and knowledge accumulation in PGs, and if a positive impact on clinical trajectory can be found. In addition, while reduced information-seeking has been observed in both behavioral [11] and substance addiction [25], and while behavioral cognitive and neural similarities are often observed between these two disorders [34], our study remains mute about whether this dissociation is a common code for addiction, or whether it is instead the only key to behavioral addiction. Further work is needed to test whether our findings can be generalized to other addiction types.

Concerning healthy information-seeking, our results show a more nuanced view over information-seeking under repeated choices (or directed exploration [4]). While in previous RL models, directed exploration was modeled as knowledge or uncertainty parameter added to the value function [4, 6, 8, 52, 53], here we were able to dissociate the contribution of novelty-seeking and general information-seeking to human exploration. We observe that a novelty bonus and general information can play dissociable roles, with potentially different implications for different decision-making scenarios or exploratory phases. Our findings, therefore, strengthen the view of exploration as a multifaceted and sophisticated process [4, 53]. Moreover, our results replicate previous findings that assign different behavioral roles and neurocognitive mechanisms to informative and undirected components of exploration [4, 6, 9, 54–57]. Indeed, PGs displayed reduced directed exploration (defined here as choosing the most informative option—the novel option) but not undirected (or random) exploration (both in terms of softmax parameter and exploratory choices made in the Equal Information condition).

Altogether, our findings extend the scientific understanding of human information-seeking in healthy and addictive behaviors. HCs and PGs showed distinct information-seeking modes. Healthy information-seeking appears primarily motivated by novelty, while PGs’ information-seeking is characterized by reduced novelty and increased knowledge accumulation. Our results suggest that the expression of novelty-seeking behavior might be a potential predictor of human wellbeing, and the expression of altered information-seeking patterns is a potential marker of behavioral addiction. Methodologically, this work offers promising novel experimental and computational approaches for studying the mechanisms underlying information-seeking under repeated choices in both healthy and pathological populations.

## Supplementary information

Supplementary material
