# Supplementary material for: Distinct motivations to seek out information in healthy individuals and problem gamblers
Source: Transl Psychiatry. 2021 Jul 26;11:408. doi: 10.1038/s41398-021-01523-3 (PMC8313706; doi:10.1038/s41398-021-01523-3)
Supplement: Supplementary file 1 — Supplementary material [file 41398_2021_1523_MOESM1_ESM.docx]

**SUPPLEMENTARY MATERIAL**

**Distinct motivations to seek out information in healthy individuals and problem gamblers**

**Irene Cogliati Dezza^1,2,3,6*^, Xavier Noel^4^, Axel Cleeremans^1^, Angela J. Yu^5*^**

^1^Centre for Research in Cognition & Neurosciences, ULB Neuroscience Institute, Université Libre de Bruxelles, Belgium

^2^Department of Experimental Psychology, Faculty of Brain Sciences, University College London, London, United Kingdom

^3^The Max Planck UCL Centre for Computational Psychiatry and Ageing Research, University College London

^4^Faculty of Medicine, Université Libre de Bruxelles, Belgium

^5^ Department of Cognitive Science, University of California San Diego, United States

^6^ Department of Experimental Psychology, Ghent University, Belgium

*corresponding authors

**SUPPLEMENTARY METHODS**

**Clinical and demographic characteristics**

Participants were recruited by placing ads on the university campus, on Facebook groups and in gambling locales. Inclusion/exclusion criteria were examined the day before the experiment by conducting a short telephone interview as well as on the day of the experiment by filling self-reported questionnaires presented in a random order during the last part of the experimental session. The telephone interview was adopted as pre-screening for both PGs and HCs. We specifically asked for information concerning age, gender, frequency of gambling per week (for PGs) or last gambling experience (for HCs), consumption of alcohol or other substances (including legal and illegal drugs) per week , inability to stop drinking alcohol, undergoing psychological treatments, and possible brain surgeries underwent in the past. We interviewed about N=60 gamblers. Gamblers who met the criteria were then invited to take part to the experiment (N=40). We then took the demographics of the gambling group (gender and age) and we set them as criteria for selecting the control group (alongside with no gambling experience in the year before the study, no sign of excessive use of alcohol or use of substances, psychological treatments, possible brain surgeries etc.). We interviewed about the same number of participants as for the gambling group. More than half of the sample was rejected because of gender (as the gambling group was mostly composed of males) and age (gamblers were quite old compared to usual undergraduates or master students who take part to psychological experiments at the University). In the following two sections, we describe the clinical and demographic characteristics of PGs and HCs.

*Problem gamblers*

Gambling severity was evaluated using the Canadian Problem Gambling Index (CPGI ^1^). Eight gamblers were classified as low level of problem gambling with 1$\leq$ GPCI $\leq$3, thirteen gamblers with moderate level of problem gambling (leading to some negative consequences; 4$\leq$GPCI $\leq$7), and nineteen as exhibiting pathological problem gambling (with negative consequences and possible loss of control; GPCI$\geq$8). We also interviewed participants using DSM-V (French translation) and we observed that 52.4% of PGs met the DSM-V criteria for gambling disorder ^2^. The relatively low level of gambling addiction presented in this population is the result of including only participants who showed no co-morbidities with substance abuse or alcohol use disorder. Specifically, to be able to tell apart effects of addictive behaviors *per se* on decision-making from effects of long-term intake of chemical compound, we tested PGs with no use (N= 31, Drug Abuse Screening Test ^3^- DAST =0) or non-problematic use (N=9, DAST =1) of legal and illegal substances and with absence of alcohol addiction (Alcohol Use Disorders Identification Test ^4^- AUDIT- <12 in men and AUDIT < 11 in women, M = 4.625, SD = 3.868; N=30 did not show any misuse of alcohol AUDIT< 8). We also controlled for smoking addiction using the Fagerström Test for Nicotine Dependence- FTND ^5^. Seven participants reported to smoke, but only 2 were classified with a mid-dependence and 2 with a weak-dependence, the other 3 were not dependent. Given that the main statistical results remained unchanged after removing those participants, we decided to include them in all the analyses. Additionally, to avoid the scenario that participants under psychological treatment may have developed a certain type of cognitive strategy over their decision processes, we included only participants who were not undergoing or seeking for psychological treatment. Moreover, we only included regular gamblers that were gambling at least once per week. Finally, we recruited both strategic PGs (sport betting, poker, black jack; N=22) and non-strategic PGs (bingo, lotto, slot machine, roulette; N=18) ^6^. Given that no behavioral difference was found between the two sub-types (in line with ^7^), we combined strategic and non-strategic gamblers in the same gambling group in all analyses reported in this manuscript.

*Healthy controls*

The inclusion criteria for the HC group were as follow: CPGI=0 and no gambling experience in the past 12 months. 40% of control participants reported to have gambled in the past years, whereas the rest of the group reported to have never gambled in their life. As for the problem gambling group, we only included participants who scored DAST < 2 (with 17 subjects DAST = 0) and AUDIT < 12 (for the men), 11 (for the women) (with 17 subjects scored AUDIT< 8; M = 5.3, SD = 3.1). Three participants reported to smoke, two of them showed no sign of addiction (FTND = 0 ; 2) and one showed mid-level of addiction (FTND = 7). Removing this participant did not change the main statistical results, therefore the participant was included in all the analyses.

**Behavioral Task**

To study information-seeking behavior under repeated choices, we adopt a modified version of a popular task (i.e., the multi-armed bandit) often used to study sequential learning and decision-making behavior. In the bandit task, the decision-maker must make repeated choices among options characterized by initially unknown reward distributions. Each choice can be driven either by a more myopic desire to maximize immediate gain (based on knowledge gained from previous choices and outcomes) or by a more long-term goal of being more informed about all the options. In these repeated scenarios, however, the more the decision-maker tends to choose the most rewarding options, the more those rewarding options tend to be (anti-) correlated with the amount of (remaining) information that can be obtained ^8^ ^9^. Accordingly, these classical decision-making tasks make it difficult to quantify exactly how much reward and information each contribute independently to choices ^9^. Here, we therefore adopt a novel variant of the bandit task ^10^, inspired by ^9^, which has an initial phase of forced choices that carefully controls for reward and information associated with each option. In particular, the influence of reward and information on choices is orthogonalized in the first free-choice trial (since after receiving the feedback on the first free-choice trial, subjects tend to choose the more rewarding options more often, thus reward and information become anti-correlated). Adding a forced-choice task before the actual decision task allows to control for available information and the reward magnitude associated with each option (i.e., options associated with the lowest amount of information were least associated with experienced reward values)^9^. This procedure allows to dissociate between information-driven exploration and undirected exploration. For instance, in the unequal sampling condition, the deck never selected during the forced choice task has highest informative value (it is completely unknown to participants) but it has no reward value associated with. By choosing that deck, participants are engaging in information-driven exploration. On the contrary, in the equal information condition, no differences are observed in terms of information. Therefore, whenever participants choose to explore, this strategy is not driven by an informative drive but only by decision noise^9^.

Contrary to our previous versions of this task ^10^ ^11^, in half of the games of the equal reward-equal information condition, we introduced an unusually high reward outcome (with respect of the deck mean in that game) for a specific option (e.g., 90 points) the first time that this option was selected in the forced-choice task (subsequently the mean of the deck was set to its original value). This manipulation was introduced as a control condition in order to test whether gamblers’ perseverate in choosing a generally poor option that they initially have a good experience with (the ‘big win’ hypothesis for gambling addiction ^12^).

Prior to beginning the main experiment, participants were told that during the forced-choice task, they may sample options at different frequencies, and that the decks of cards did not change during each game, but were replaced by new decks at the beginning of each new game. However, they were not informed of the details of the reward manipulation or the underlying generative distribution adopted during the experiment.

**Computational Modelling**

In this section, we provide details on the RL models adopted in this study.

*Standard RL model*

The standard RL (sRL) model learns reward values on each trial using the delta learning rule^13^:

$$Q_{t+1, j}\left( c \right)= Q_{t,j}\left( c \right)+\alpha\times\delta_{t,j}$$

where, $\delta_{t,j}= R_{t,j}(c)-Q_{t,j}\left( c \right)$ (S1)

where $Q_{t,j}\left( c \right)$ is the expected reward value for trial *t* and game *j* and $\delta_{t,j}$is the *prediction error*, which quantifies the discrepancy between the previous predicted outcome $Q_{t,j}\left( c \right)$ and the actual outcome $R_{t,j}$obtained at trial *t* and game *j*. Since participants were told that games were independent from one another, $Q_{0}$is initialized at the beginning of each game to the global estimate of the expected reward values for each deck. We previously showed that this initialization was better able to capture healthy participants’ behaviour than learning $Q_{0}$ on a trial-by-trial basis ^10^. Next, a choice is made by entering expected reward values into the softmax function ^14^, as follows:

$P(c/Q_{t, j}\left( c_{i} \right)) =\frac{exp(\beta\times Q_{t, j}\left( c \right))}{\sum_{i} exp(\exp\beta\times Q_{t, j}(c_{i}))}$ (S2)

where $\beta$ is the inverse temperature that determines the degree to which choices are randomized by decision stochasticity (or choice variability).

*Knowledge RL model*

As sRL, the knowledge RL (kRL) model learns reward values using Eq. S1 but it additionally integrates information obtained from each deck into the value function:

$V_{t, j}\left( c \right)= Q_{t+1, j}\left( c \right)+I_{t, j}\left( c \right)* k$ (S3)

where, $i_{t,j}\left( c \right)=\left\{ \begin{aligned} 0, choice\neq c \\ 1, choice=c \end{aligned} \right.$

κ modulates the importance of information relative to experienced reward. With large κ the model favors already experience decks, while with negative values of κ the model explores new information more frequently. A choice is made by entering choice values $V_{t, j}\left( c \right)$into Eq. S2.

*Novelty-knowledge RL model*

As the above models, the novelty-knowledge RL (*nkRL*) model learns reward values using Eq. S1. And, it additionally integrates information into the value function as kRL. However, as described in the main text, nkRL computes information as a sum of knowledge term and novelty term resulting in the following value function:

$V_{t, j}\left( c \right)= Q_{t+1, j}\left( c \right)+\sum_{1}^{t} i_{t,j}\left( c \right)* k+1_{\mathrm{novel}}* \nu$ (S4)

A choice is made by entering choice values $V_{t, j}\left( c \right)$into Eq. S2.

*Leaky nkRL model*

The leaky nkRL model learns reward values using Eq. S1 and it integrates both knowledge and novelty term into the value function as nkRL. However, in leaky nkRL each bit of new information is integrated in a leaky fashion as follow:

$V_{t, j}\left( c \right)= Q_{t+1, j}\left( c \right)+\sum_{1}^{t} i_{t,j}\left( c \right)* k+1_{unseen}* \nu$ (S5)

where, $i_{t,j}\left( c \right)=\left\{ \begin{aligned} 0, choice\neq c \\ 1*\lambda, choice=c \end{aligned} \right.$ (S6)

*Gamma nkRL model*

The gamma nkRL (gnkRL) model learns reward values using Eq. S1, and it integrates both knowledge and novelty term into the value function as nkRL. However, gnkRL allows a non-linear integration of information:

$V_{t, j}\left( c \right)= Q_{t+1, j}\left( c \right)+\left( \sum_{1}^{t} i_{t,j}\left( c \right) \right)^{\gamma}* k+1_{unseen}* \nu$ (S7)

γ defines both the degree of non-linearity in the amount of observations obtained from options after each observation and its related importance. Under high γ the information already gained is highly relevant, whereas the information to be acquired is less relevant or penalized. γ is constrained to be > 0.

*Model fitting and Model selection*

The models’ parameters were estimated by fitting the model to trial-by-trial participants’ free choices (~600 choices for each subject). The fitting procedure was performed using MATLAB function *fminsearchbnd* and iterated for 15 randomly chosen multiple starting points in order to minimize the chance of finding a local optimum instead of a global one. The fitting procedure was validated by running a recovery analysis: the model was simulated on the task using the retrieved parameter estimates to generate synthetic behavioral data and then the fitting procedure was applied to the synthetic data in order to check whether previously estimated parameters were indeed recovered ^15^ (**Figure S1**). For model comparisons, negative log likelihoods obtained during the fitting procedure were used to compute model evidence (the probability of obtaining the observed data given a particular model). We adopted an approximation to the (log) model evidence, namely the Bayesian Information Criterion (BIC) ^16^ and we compared its estimate across different models (fixed-effect comparison). Additionally, we used random-effects procedure to perform Bayesian model selectin at group level ^17^. In order to inspect the fitting procedure for overfitting we adopted cross validation procedure ^18^. We fitted the model to 70% of the trials and we tested its ability to predict choices on future data (30% of the trials) compared to a simpler nested model. We then adopted the likelihood ratio test to determine if the better fit of complex model was due to noise captured in the data.

**Statistical analysis**

Statistical analysis was performed using RStudio (<https://www.rstudio.com/>). When violations of parametric tests were indicated, non-parametric tests were performed. *P*-values < .05 were considered significant.

**SUPPLEMENTARY RESULTS**

**PGs and HCs show comparable choice behavior when choices are equally informative**

The reduced novelty-seeking behavior in PGs found above could either be due to a specific decrease in the valuation of novelty, or a relative and general increase in the valuation of reward. To examine this, we compare the two groups’ behaviors in the Equal Information condition, in which the options have been sampled equal number of times and thus equally informative – any systematic difference in reward-seeking behavior here would be attributable specifically to reward and not influenced by general information or novelty. Again, we focus on the first free-choice trial, where there is no confound between reward and information. We classified choices as *reward-seeking* when choosing the option associated with the highest amount of points and *undirected exploration* otherwise. We then entered these values into a mixed effects logistic regression predicting choice type (reward-seeking, undirected exploration) with group (PGs, HCs) and reward condition (Low Reward, High Reward) and their interaction as fixed effects and subjects as random intercepts (1|Subject). This model had lower BIC (5956.2) compared to a model with random intercepts and slopes (BIC = 5977.7). Replicating previous studies using the same experimental design on healthy participants ^10^ ^11^, we found a fixed effect of reward (beta coefficient = -0.351 ± 0.109 (SE), z = -3.23, *p* < 10^-2^), with undirected exploration lower in the Low Reward condition. The fact that low reward enhances novelty-seeking but reduces undirected exploration suggests that these are dissociable exploratory drives in the brain with dissociable neural substrate ^11,19^. Most importantly, the effect of group (beta coefficient = 0.113 ± 0.191 (SE), z = 0.589, *p* = 0.556) and the interaction between group and reward (beta coefficient = -0.016 ± 0.135 (SE), z = 0.116, *p* = 0.908) were not significant. The results from the current analysis, along with those from the previous analysis, suggest that the reduced novelty-seeking behavior in PGs is specific to novelty and not an indirect consequence of greater valuation of immediate reward in general (**Figure 2b**).

**Novelty-familiarity shift is absent in PGs**

Here, we examine choices made by participants across the entire set of free choice trials in the Unequal Information conditions. We classified a choice as an *informative choice* when subjects chose the option sampled the least number of times thus far, and *familiar choice* when they chose the option sampled the most number of times so far. We calculated the number of trials in which each choice was made and divided them by the total number of informative and familiar trials to obtain their relative frequencies (i.e. we exclude trials in which the subject chose the option that was neither most familiar nor most informative). We then entered those values into a mixed effects logistic regression predicting choices (informative, familiar) from group (PGs, HCs) and trial (1,2,3,4,5,6), and their interaction as fixed effects and subjects as random intercepts (1|Subject; this model had lower BIC compared to a model with random intercepts and slopes). This revealed a fixed effect of group (beta coefficient = 0.546 ± 0.203 (SE), z = 2.69, *p* = 0.007) and fixed effect of trials (beta coefficient = 0.419 ± 0.021 (SE), z = 19.58, *p* < 10^-3^) as when both equal information and unequal information games were included. However, narrowing the analysis to the Unequal Information condition also revealed an interaction effect between group and trial (beta coefficient = -0.06 ± 0.027 (SE), z =-2.23, *p* = 0.026), such that the shift in preference from more informative options early on in the free-choice task to more familiar options later on was smaller in PGs than HCs. To better understand this interaction, we compared subjects’ tendency to choose the most informative versus most familiar option on the first and sixth trial of the free choice task. We found that control subjects preferred novel options (M= 0.641, SD= 0.257) over familiar options (M= 0.359, SD= 0.257; *p* = 0.002; **Figure 2d**) on trial 1, but reversed preferences to prefer familiar options over informative options on trial 6 (M = 0.705, SD = 0.121, *p* < 10^-3^). In contrast, PGs preferred novel options (M= 0.51, SD = 0.222) and familiar options (M= 0.49, SD = 0.222) equally on trial 1, but strongly preferred familiar options (M= 0.807, SD = 0.149, *p* < 10^-3^) over informative options (M= 0.193, SD = 0.149) on trial 6 (**Figure 2e)**. Thus, the “novelty-familiarity” shift was apparent in HCs but absent in PGs.

**Model comparison**

We first examine whether our nkRL model was better able to explain participants’ behavior compared to a standard RL (sRL) model ^13^ -where only reward predictions influence choices- and, to a knowledge RL (kRL) model ^10^ –which combines both reward and knowledge associated with options without explicitly decomposing information into novelty and general information. We chose kRL as example of unitary models (i.e., information is not decomposed in different drives) because previous researches showed that kRL was better able to explain human behavior in our behavioral task compared to models which update learning rate as number of observations (e.g., Kalman filter, ^10^). We fit the 4 models to participants’ data and we computed model evidence as approximation of –BIC/2. We removed two subjects (one from each group) for bad fitting. These subjects were removed from all model-based analyses reported in the main text. We then utilized Bayesian Model Selection ^17^ to compare the 3 models. We found nkRL model was the best model for predicting choice behavior in both HCs (xp_nkRL_=1, BIC_nkRL_=18065.6; xp_kRL_=0, BIC_kRL_= 18918; xp_sRL_=0, BIC_sRL_= 19407; **Figure 3a**) and PGs (xp_nkRL_=0.877, BIC_nkRL_= 33577.2; xp_kRL_=0.058, BIC_kRL_= 35080.1; xp_sRL_=0.065, BIC_sRL_= 35683.9; **Figure 3b**). Next, we asked whether participants were integrating complete information into the value function, as predicted by nkRL, or instead information was integrated in a leaky fashion. We implemented a new model (leaky nkRL) where each sample of information integrates as 1*λ, where λ is the leaky integration parameter. Model comparison showed that nkRL model was better able to explain both PGs (xp_nkRL_= 0.9999, BIC_nkRL_= 33577.2; xp_leaky_nkRL_=0.0001, BIC _leaky_nkRL_=33795.2; **Figure 3b**) and HCs’ choices (xp_nkRL_= 1, BIC_nkRL_=18065.6; xp _leaky_nkRL_=0, BIC _leaky_nkRL_=18188.7; **Figure 3a**). Lastly, we examined how information affects choice values. It may be the case that at least for certain situations (as in the present task) in which only a few samples of each option are available, additional observations may provide a non-constant amount of information and therefore they may scale choice value in a sub or super-linearly fashion. We compared nkRL, where information is measured linearly in the number of observations, with a model that permits the integration of information sub- or super-linearly (gnkRL). Model comparison showed that nkRL model was better able to explain both PGs (xp_nkRL_=1, BIC_nkRL_= 33577.2; xp_gnkRL_=0, BIC_gnkRL_= 33703.9; **Figure 3b**) and HCs’ choices (xp_nkRL_=1, BIC_nkRL_= 18065.6; xp_gnkRL_=0, BIC_gnkRL_= 18137.4; **Figure 3a**). Thus, we found nkRL to be the best-fitting model among all those that we considered.

**Parameter recovery**

We performed a parameter recovery analysis to estimate the degree of accuracy of the fitting procedure . To do so, we simulated data from nkRL using the parameters obtained from the fitting procedure (*true parameters*), and we fit the model to those simulated data to obtain the estimated parameters (*fit parameters*). We then ran a correlation for each pair of parameters ^15^ (**Figure S1**). This revealed high correlation coefficients for alpha (r_HCs_ = 0.8, *p*_HCs_ < 10^-3^; r_PGs_ = 0.9, *p*_PGs_ < 10^-3^), knowledge (r_HCs_ = 0.9, *p*_HCs_ < 10^-3^; r_PGs_ = 0.6, *p*_PGs_ < 10^-3^) and novelty (r_HCs_= 0.98, *p*_HCs_ < 10^-3^; r_PGs_ = 0.8, *p*_PGs_ < 10^-3^). The beta parameter showed high correlation coefficient in PGs (r = 0.9, *p* < 10^-3^). In HCs one participant showed bad fitting while the rest of the group showed high correlation coefficient (r = 0.97, *p* < 10^-3^). We removed this participant during the comparison of the beta parameter.

**Simulations nkRL with random parameters**

In this section, we report the result of the simulation of the nkRL model with random parameters to better understand the effect of novelty on choice behavior. We simulated nkRL with High Novelty and Low Novelty parameter. In each set of simulations, nkRL was simulated 100 times. In High Novelty, the averaged values of the parameters were as follow: alpha (M = 0.513, SD = 0.315), beta (M= 0.52, SD = 0.283), knowledge (M = 0.493, SD = 0.288), novelty (M = 41.38, SD = 11.31). In Low Novelty, we used the following averaged values: alpha (M = 0.519, SD = 0.304), beta (M = 0.51, SD = 0.293), knowledge (M = 0.479, SD = 0.282), novelty (M = -0.839 SD = 0.584). We then classified model choices in reward-seeking (when the model chooses the experienced decks with the highest average of points regardless of the number of times that deck had been selected during the forced-choice task) and novelty-seeking (when the model selects the option never sampled during the forced-choice task) in the first free-choice trial of the unequal information condition. As shown in **Figure S2a**, under Low Novelty the model increases reward-seeking at the expense of novelty-seeking as observed in PGs (**Figure 2a**). Next, we calculated the number of trials in which the model was choosing the partially informative option (seen twice) in the first free-choice trials of the unequal information condition and we averaged those estimates across the trials in which the model engages in information-seeking (novelty-seeking + general information-seeking). As shown in **Figure S2b**, under Low Novelty the model increases the selection of options selected twice during the forced-choice task (general information-seeking) at the expense of novel options as observed in PGs (**Figure 2e**).

**Personality traits**

In this section, we explore the individual differences between PGs and HCs to investigate whether personal traits could explain the behavioral differences observed throughout our analyses. We focus on intolerance of uncertainty (EII ^20^), impulsivity (UPPS-P ^21^), sensation-seeking (SSS ^22^), and sensitivity to punishment and reward (SPSRQ ^23^). Comparisons between HCs and PGs revealed no differences in the scores obtained from EII (*p* = .785, BF_01_ = 3.61), UPPS-P (*p* = .217, BF_01_ = 1.89), SSS (*p* = .483, BF_01_ = 3.02), and SPSRQ (sensitivity to reward *p* = .399, BF_01_ = 2.81; sensitivity to punishment *p* = .266, BF_01_ = 2.4), suggesting that the behavioral alterations observed in PGs are unlikely to be explained as differences in terms of personality traits (or in some cases there was not substantial evidence in favor of the alternative hypothesis). These results appear to suggest that reduced novelty-seeking in PGs may relate to a process or mechanism that is independent from individual subjective preferences toward uncertainty, sensation-seeking, or punishment and reward sensitivity.

**The ‘big win’ hypothesis**

The results reported in this study showed that PGs reduced novelty-seeking behaviors as a consequence of a failure to represent or incorporate a novelty bonus. However, these parametric alterations might have been confounded by the inability of PGs of moving away from an option after experiencing fairly positive outcomes in the past, i.e., the ‘big win’ hypothesis. To better investigate this point, we computed the empirical probability of choosing an option associated with an unusually high score (“big win” options) when first selected in the forced-choice task. A two-sample t test showed no differences in the probability of choosing the “big win” option in PGs (M = 0.607 SD = 0.187) compared to HCs (M = 0.596 SD = 0.144), *p* = .798 suggesting that PGs’ choice behavior was not driven by the persistence in choosing options associated with unusually good outcomes in the past.

**SUPPLEMENTARY REFERENCES**

1 Ferris, J. & Wynne, H. The Canadian problem gambling index: Final report. (Canadian Centre on Substance Abuse., Ottawa (ON)

, 2001).

2 APA. *Diagnostic and Statistical Manual of Mental Disoders*. (American Psychiatry Publishing, 2013).

3 Skinner, H. A. The Drug Abuse Screening Test. *Addict Behav* **7**, 363-371 (1982).

4 Babor, T. F., de la Fuente, J. R., Saunders, J. & Grant, M. (World Health Organization).

5 Fagerström, K. O. Measuring degree of physical dependence to tobacco smoking with reference to individualization of treatment. *Addictive Behaviors*, 235–241 (1978).

6 Lorains, F. K. *et al.* Strategic and non-strategic problem gamblers differ on decision-making under risk and ambiguity. *Addiction* **109**, 1128-1137, doi:10.1111/add.12494 (2014).

7 Grant, J. E., Odlaug, B. L., Chamberlain, S. R. & Schreiber, L. R. Neurocognitive dysfunction in strategic and non-strategic gamblers. *Prog Neuropsychopharmacol Biol Psychiatry* **38**, 336-340, doi:10.1016/j.pnpbp.2012.05.006 (2012).

8 Hertwig, R. & Erev, I. The description-experience gap in risky choice. *Trends Cogn Sci* **13**, 517-523, doi:10.1016/j.tics.2009.09.004 (2009).

9 Wilson, R. C., Geana, A., White, J. M., Ludvig, E. A. & Cohen, J. D. Humans use directed and random exploration to solve the explore-exploit dilemma. *Journal of experimental psychology. General* **143**, 2074-2081, doi:10.1037/a0038199 (2014).

10 Cogliati Dezza, I., Yu, A. J., Cleeremans, A. & Alexander, W. Learning the value of information and reward over time when solving exploration-exploitation problems. *Sci Rep* **7**, 16919, doi:10.1038/s41598-017-17237-w (2017).

11 Cogliati Dezza, I., Cleeremans, A. & Alexander, W. Should we control? The interplay between cognitive control and information integration in the resolution of the exploration-exploitation dilemma. *Journal of experimental psychology. General*, doi:10.1037/xge0000546 (2019).

12 Kassinove, J. I. & Schare, M. L. Effects of the "near miss" and the "big win" on persistence at slot machine gambling. *Psychol Addict Behav* **15**, 155-158 (2001).

13 Rescorla, R. A. & Wagner, A. R. A theory of Pavlovian conditioning: Variations in the effectiveness of reinforcement and nonreinforcement. *Classical conditioning: Current research and theory*, 64-99 (1972).

14 Bishop, C. M. *Pattern recognition and machine learning*. (2006).

15 Wilson, R. C. & Collins, A. G. Ten simple rules for the computational modeling of behavioral data. *Elife* **8**, doi:10.7554/eLife.49547 (2019).

16 Schwarz, G. Estimating the dimension of a model. *Ann. Stat* **6**, 461-464 (1978).

17 Stephan, K. E., Penny, W. D., Daunizeau, J., Moran, R. J. & Friston, K. J. Bayesian model selection for group studies. *Neuroimage* **46**, 1004-1017, doi:10.1016/j.neuroimage.2009.03.025 (2009).

18 Daw, N. D. in *Affect, Learning and Decision Making, Attention and Performance XXIII* (eds E.A. Phelps, T.W. Robbins, & M. Delgado) (Oxford University

Press, 2009).

19 Zajkowski, W. K., Kossut, M. & Wilson, R. C. A causal role for right frontopolar cortex in directed, but not random, exploration. *Elife* **6**, doi:10.7554/eLife.27430 (2017).

20 Buhr, K. & Dugas, M. J. The Intolerance of Uncertainty Scale: psychometric properties of the English version. *Behav Res Ther* **40**, 931-945 (2002).

21 Billieux, J. *et al.* Validation of a short French version of the UPPS-P Impulsive Behavior Scale. *Compr Psychiatry* **53**, 609-615, doi:10.1016/j.comppsych.2011.09.001 (2012).

22 Zuckerman, M. & al., e. Development of a sensation-seeking scale. *Journal of Consulting Psychology* (1964).

23 Torrubia, R., Avila, C., Moltó, J. & Caseras, X. The sensitivity to punishment and sensitivity reward questionnaire (SPSRQ) as a measure of Gra's anxietyand impulsivity dimensions. *Personality and Individual Differences* **31**, 837-862 (2001).

**SUPPLEMENTARY FIGURES**

**Figure Captions**

**Figure S1. *Parameter Recovery*.** Correlation between true and fit parameters for nkRL model. True parameters are those recovered during the fitting procedure, while fit parameters are those recovered after fitting the model to synthetic data (obtained by simulating nkRL with parameters estimated in the two groups).

**Figure S1. *nkRL simulations with random parameters*.** Under Low Novelty the model frequently engages in reward-seeking (**a**) and in general information-seeking (**b**).

**Figure S1.**


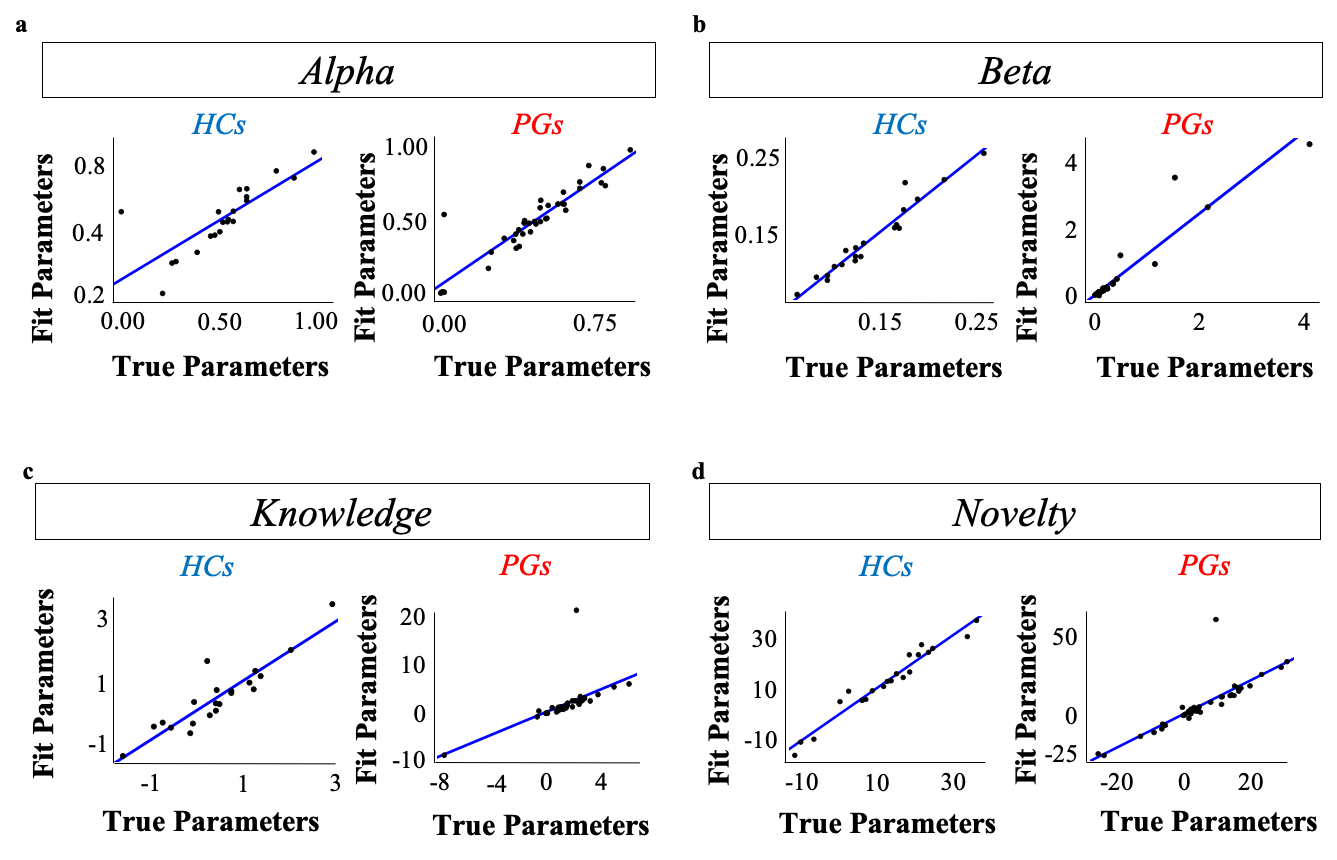


**Figure S2.**

**
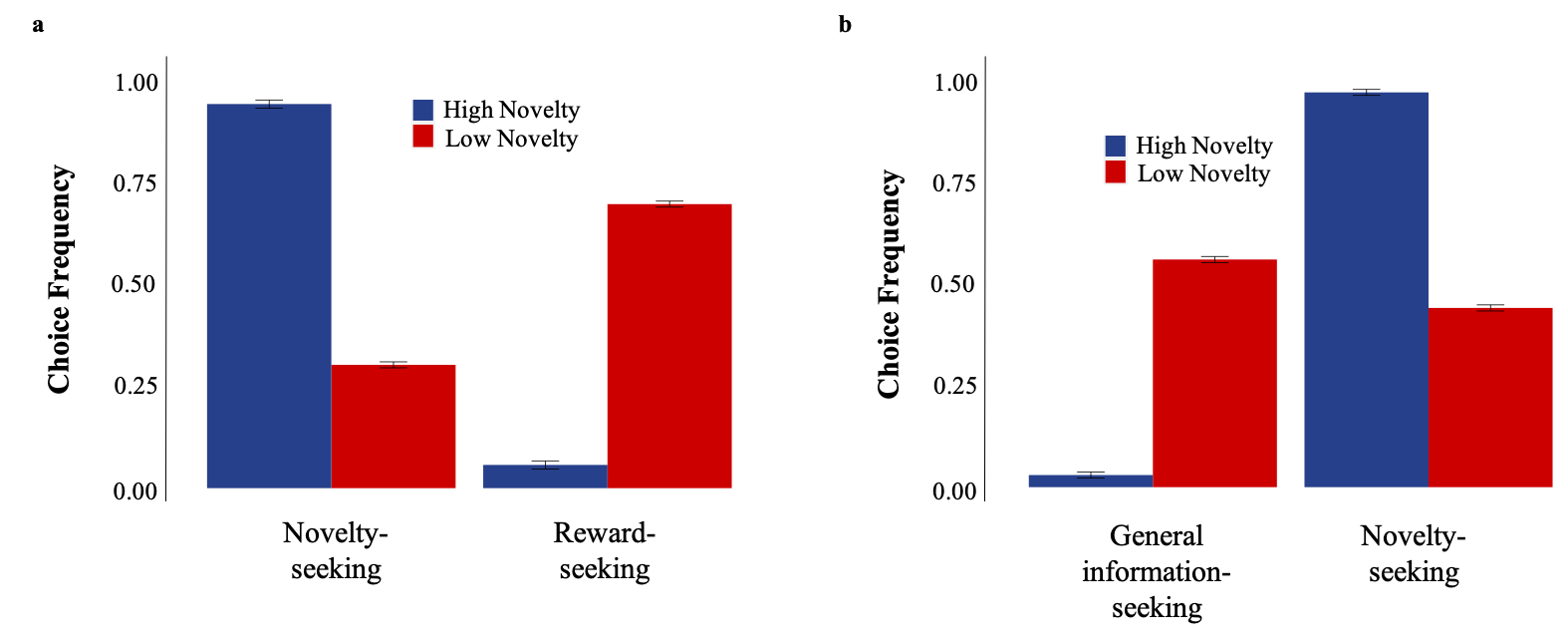
**
